# Evaluation of radiomics feature stability in abdominal monoenergetic photon counting CT reconstructions

**DOI:** 10.1038/s41598-022-22877-8

**Published:** 2022-11-15

**Authors:** Hishan Tharmaseelan, Lukas T. Rotkopf, Isabelle Ayx, Alexander Hertel, Dominik Nörenberg, Stefan O. Schoenberg, Matthias F. Froelich

**Affiliations:** 1 Department of Radiology and Nuclear Medicine, University Medical Center Mannheim, Heidelberg University, Theodor-Kutzer-Ufer 1-3, 68167 Mannheim, Germany; 2 Department of Radiology, German Cancer Research Center, Im Neuenheimer Feld 230, 69120 Heidelberg, Germany

**Keywords:** Computational models, Data processing, Image processing, Software, Medical imaging

## Abstract

Feature stability and standardization remain challenges that impede the clinical implementation of radiomics. This study investigates the potential of spectral reconstructions from photon-counting computed tomography (PCCT) regarding organ-specific radiomics feature stability. Abdominal portal-venous phase PCCT scans of 10 patients in virtual monoenergetic (VM) (keV 40–120 in steps of 10), polyenergetic, virtual non-contrast (VNC), and iodine maps were acquired. Two 2D and 3D segmentations measuring 1 and 2 cm in diameter of the liver, lung, spleen, psoas muscle, subcutaneous fat, and air were obtained for spectral reconstructions. Radiomics features were extracted with pyradiomics. The calculation of feature-specific intraclass correlation coefficients (ICC) was performed by comparing all segmentation approaches and organs. Feature-wise and organ-wise correlations were evaluated. Segmentation-resegmentation stability was evaluated by concordance correlation coefficient (CCC). Compared to non-VM, VM-reconstruction features tended to be more stable. For VM reconstructions, 3D 2 cm segmentation showed the highest average ICC with 0.63. Based on a criterion of ≥ 3 stable organs and an ICC of ≥ 0.75, 12—mainly non-first-order features—are shown to be stable between the VM reconstructions. In a segmentation-resegmentation analysis in 3D 2 cm, three features were identified as stable based on a CCC of > 0.6 in ≥ 3 organs in ≥ 6 VM reconstructions. Certain radiomics features vary between monoenergetic reconstructions and depend on the ROI size. Feature stability was also shown to differ between different organs. Yet, glcm_JointEntropy, gldm_GrayLevelNonUniformity, and firstorder_Entropy could be identified as features that could be interpreted as energy-independent and segmentation-resegmentation stable in this PCCT collective. PCCT may support radiomics feature standardization and comparability between sites.

## Introduction

Radiomics analyses are widely used in radiological research and promise to deliver quantitative non-human-readable information to address diagnostic and prognostic challenges in modern medicine. Their potential lies in the ability to extract a vast number of features that can help to tackle such challenges in radiology. Radiomics in many cases has even outperformed traditional clinical scores^1,2^. Gaining quantifiable objective data on regions of interest provides additional information for the increasingly big data-oriented developments in healthcare that can support existing decision-making algorithms.

The methodology of radiomics has most prominently been applied in oncologic studies^3–6^. For example, radiomics-based models were able to differentiate malignant and benign early-stage lung nodules in computed tomography^7^. However, radiomics has also been shown to be powerful in cardiovascular imaging^8,9^, chronic diseases such as lung fibrosis^10,11^, urologic diseases such as kidney stones^12^, and infectious diseases such as COVID-19^13,14^ or mycobacterial lymphadenitis^15^.

Despite these auspicious research results, the transition to the application of radiomics in clinical practice has not been realized yet. One of the main reasons for this is the issue of radiomics feature reproducibility. Radiomics features comparability is impacted by a broad range of factors at many points of analysis. Image acquisition settings such as the choice of the scanner, tube voltage, reconstruction kernel, choice of contrast agent, and a variety of other aspects can be a reason for diminished reproducibility^16–18^. Another component is the segmentation method (manual, semi-automatic, fully automated) which can produce different results^19^. Furthermore, it has been shown that the feature extraction software can influence the radiomics features^20^.

For computed tomography, the introduction of photon-counting detector computed tomography (PCCT) technology promises to deliver a higher spatial resolution resulting from the direct measurement of individual photons. Traditional, energy integrating detectors measure X-rays in an indirect approach, utilizing scintillator technology, and, therefore, include a separate step involving the generation of visual light, which is measured in a photodiode. In contrast, photon-counting detectors measure the photons' energy directly^21^. In addition, they enable the possibility of creating virtual monoenergetic (VM), iodine maps, and virtual non-contrast (VNC) enhanced images from the spectral input data, which was limited to dual-energy CT before the implementation of PCCT. The direct measurement process of photons may help address the topic of radiomics feature stability in CT and thus may increase their reliability. Thereby, PCCT could pave the way for the broad clinical implementation of radiomics-based methods. Yet, the radiomics feature properties in these specific PCCT reconstructions remain unidentified. Furthermore, the organ-specific properties and the influence of segmentation approaches in PCCT are still to be analyzed. Therefore, this study aimed to comprehensively evaluate radiomics feature stability in PCCT compared between different organs and with varying ROI sizes between different reconstructions, especially monoenergetic imaging reconstructions.

## Materials and methods

### Patient collective and study design

A total of 10 patients (median age 58, 7 females) that received an abdominal photon-counting computed tomography (NAEOTOM Alpha; Siemens Healthcare GmbH, Forchheim, Germany) examination between December 2021 and March 2022 at Mannheim University Medical Center were included in this prospective study.

The study was approved by the institutional review board of the Medical Faculty Mannheim and informed consent was obtained for all patients that were included in the study. All methods were carried out in accordance with relevant regulations. The datasets generated and/or analyzed during the current study are not publicly accessible due to German medical data privacy guidelines but are available from the corresponding author on reasonable request.

**Figure 1:**
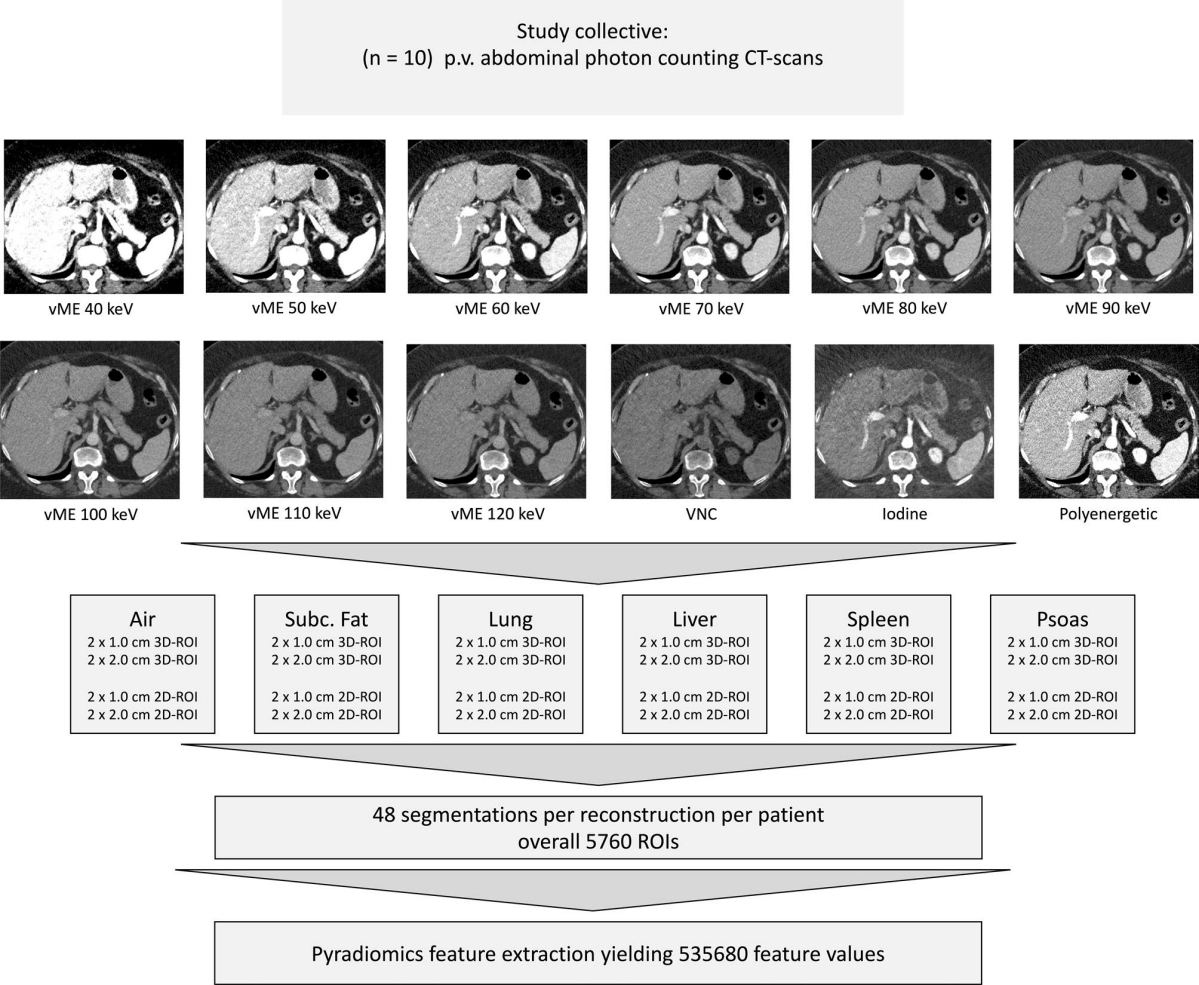
Study protocol.

Patients with an age above 18 years, CT scan local standard protocol for portal venous phase and 120 kV, tube current modulation, and presence of spectral reconstructions were included. The patients were reviewed and excluded in case of contraindicating diseases in our selected organs of interest by a clinically experienced radiologist (M.F.F.). The images were acquired in the portal venous contrast phase with weight-adapted 70–90 ml of the non-ionic iodine contrast agent Iomeprol (Imeron^®^ 350 mg iodine/ml, Bracco Imaging Deutschland GmbH, Konstanz, Germany), 120 kV tube voltage, activated CareDose 4D+, a CARE keV IQ-Level of 80, 1.5 mm slice thickness, 1.5 mm spacing, detector energy thresholds of 20/35/65/70 keV^22^, collimation of 144 × 0.4 mm, rotation time of 0.25 s, a pitch of 1.2, matrix of 512 × 512 and Qr40 kernel. The study protocol and patient characteristics are summarized in Fig. 1 and Table 1.

**Table 1:** Patient collective.

N	10
Age (mean (SD))	58 (13.27)
**Sex (%)**	
F (%)	7 (70.0)
M (%)	3 (30.0)
Effective tube current [mAs] (mean (SD))	111.8 (62.13)
CTDIvol [mGy] (mean (SD))	8.83 (4.91)
Dose length product [mGy cm] (mean (SD))	430.9 (242.54)

### Reconstruction

A software by the vendor (syngo.via VB60; Siemens Healthcare GmbH, Forchheim, Germany) was used to create iodine maps, virtual monoenergetic (from 40 to 120 keV in steps of 10 keV), polyenergetic T3D, and virtual-non contrast reconstructions from the SPP image files containing the spectral information from PCCT.

### Segmentation and feature extraction

The ROI for each tissue was segmented in 2D by a disk and in 3D by a sphere brush. The segmentations were acquired eight times per organ in total, twice with each 1 or 2 cm in maximum diameter. ROIs from the air, psoas muscle, liver, lung, spleen, and subcutaneous fat in the lower back were identified and segmented in 3D Slicer^23^ by a medical student (H.T. with 2 years of experience in segmentation). The scans and segmentations were reviewed by a clinically experienced radiology senior resident (M.F.F.). First-order, second-order gray level distribution features as gray level co-occurrence matrix (glcm), gray level size zone matrix (glszm), gray level run length matrix (glrlm), neighboring gray-tone difference matrix (ngtdm), and gray level dependence matrix (gldm) features were extracted using the Python package pyradiomics (version 3.0.1)^24^ without resampling in 3D. Shape features were not considered, as the ROI size and volume were standardized. Features were calculated with a bin width of 25 and a voxel array shift of 0. No voxel array shift was set to prevent the volume-confounding effects in our study, which also investigates different ROI sizes. The pyradiomics settings can be found in Supplemental Material S2.

### Analysis of feature stability by intraclass correlation coefficient

Features obtained according to this protocol were statistically analyzed in R^25^ and RStudio^26^. The intraclass correlation coefficient (ICC) was calculated using the package “psych” as the ICC (3,1) defined by Shrout and Fleiss, which has been applied in other radiomics feature stability studies^27,28^. ICC (3,1) is the intraclass correlation coefficient for a fixed set of raters (in this case the reconstructions), that rate each target (radiomic features) once. Each rating is a single observation and not the average of multiple observations^29^.

Using the ICC (3,1) the stability for each radiomic feature and each organ over all virtual reconstructions (keV from 40 to 120 in steps of 10 keV, VNC, and iodine maps) was calculated. The ICC was computed for each 2D and 3D segmentation as defined earlier with a maximum diameter of 1 and 2 cm. Since iodine maps—as a distinctive characteristic to the other reconstructions—only show regions perfused with the contrast medium, its influence on ICC was investigated by exclusion. To compare the impact of iodine, polyenergetic, and virtual non-contrast reconstructions, the ICC was calculated (1) with all reconstructions, (2) without iodine maps, (3) with VM reconstructions only. The approach with the highest ICC values was used for further analysis. The ICC for all lesions separated by organ was plotted in a scatter plot for approaches i-iii: Reconstruction to reconstruction feature-wise correlation was calculated and visualized in a correlation plot.

### Evaluation of feature stability by ROI size and dimensionality

The evaluation of feature stability on different ROI sizes and dimensionality was performed by comparing the average ICC for 2D-1 cm, 2D-2 cm, 3D-1 cm, and 3D-2 cm segmentations. A repeated measure one-way ANOVA analysis with a Geisser-Greenhouse correction was applied to measure the significant differences between the groups mentioned above^30^. The segmentation approach with the highest average ICC was used for further analysis.

### Identification of stable features and organ-specific comparison

Features with an excellent level of reliability in more than 3 organs were considered stable and selected. Excellent ICC was defined similarly to previous studies as above 0.75^31,32^, a good ICC level with values between 0.6 and 0.74, a fair level ranging from 0.4 to 0.59, or as poor for ICCs below 0.4. Radiomics feature stability depending on the tissue was evaluated by identifying the number of features with an excellent or good ICC value for each tissue.

### Analysis of segmentation–resegmentation stability by concordance correlation coefficient

To evaluate the reproducibility of features between different segmentations in the same organ Lin’s concordance correlation coefficient (CCC)^33^ was applied and calculated by comparing radiomics features between the two segmentations of the best segmentation mode with the highest average ICC value for every organ. CCC was defined as stable above a threshold of 0.6 in ≥ 3 organs in ≥ 6 VM reconstructions.

## Results

### Segmentation and feature extraction

**Figure 2:**
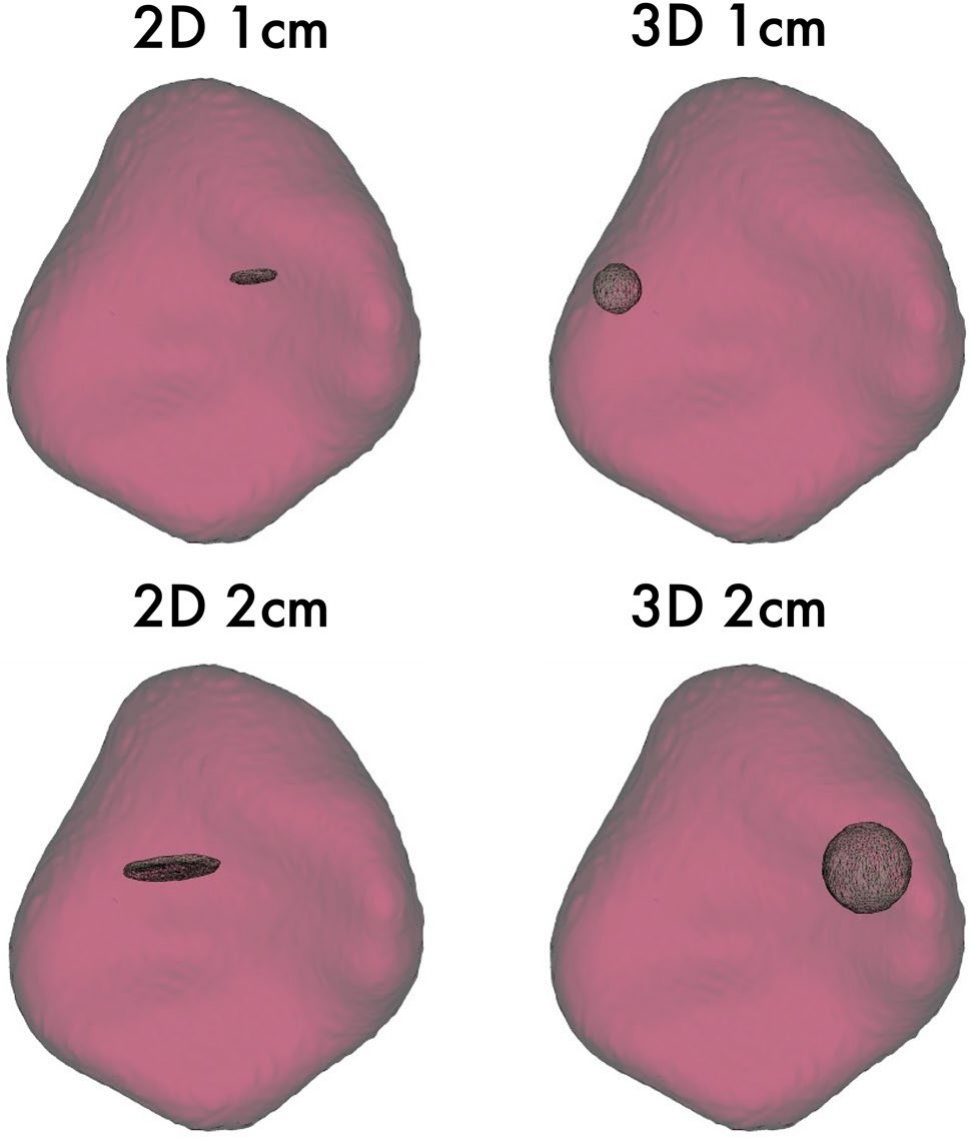
Example segmentations in a spleen with 1 cm and 2 cm maximum diameter in 2D and 3D.

The regions of interest were segmented according to the above-presented protocol. Example segmentations are displayed in Fig. 2. This resulted in 48 segmentations for each of the n = 10 patients. Features were extracted for each ROI in each of the 12 reconstructions (VM with keV 40–120 in steps of 10 keV, polyenergetic T3D image, VNC, and iodine maps). The extraction of features resulted in a total of 535.680 feature values.

### Evaluation of reconstructions and ROI size

**Figure 3:**
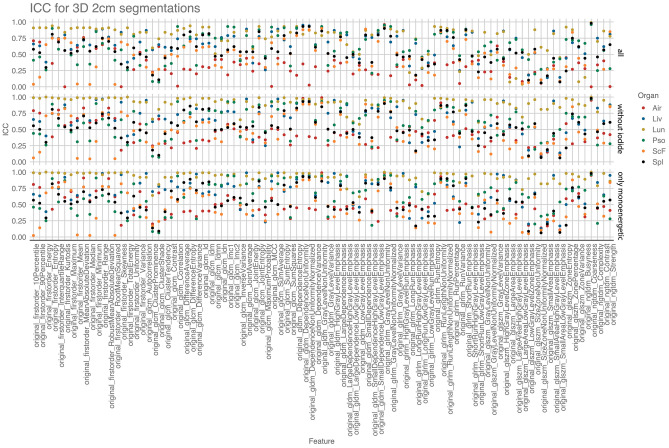
ICC values compared for (1) all reconstructions, (2) excluding iodine maps and (3) only monoenergetic reconstructions.

Following the calculation, ICC (3,1) for all lesions separated by organ was plotted in a scatter plot for the three approaches mentioned in the methods: (1) all reconstructions, (2) excluding iodine maps, and (3) only VM reconstructions (Fig. 3). Average ICC values for the approaches were obtained and compared. This showed the highest level of stability in the analysis without iodine, VNC, and polyenergetic images (3) (0.56), while the approach excluding iodine maps only (2) had an almost similar performance (0.55) (Table 2). In summary, the approach (3) focusing on VM reconstructions showed the highest degree of intraclass correlation.

**Table 2:** Average ICC values for different segmentation approaches and excluding iodine map/iodine map, virtual non contrast and polyenergetic reconstructions.

	2D-1 cm	2D-2 cm	3D-1 cm	3D-2 cm	Average ICC
(1) All reconstructions	0.43	0.53	0.51	0.56	0.51
(2) Without iodine map	0.46	0.57	0.54	0.62	0.55
(3) Monoenergetic only	0.47	0.59	0.56	0.63	0.56

Computed ICC showed a variation of the average value based on whether a 2- or 3-dimensional ROI segmentation with 1- or 2 cm was performed. The applied repeated measure one-way ANOVA analysis with a Geisser-Greenhouse correction showed significant differences (p < 0.0001) between all four segmentation groups. The 3D-2 cm segmentations achieved the highest ICC in all analysis approaches (1–3) with an average of 0.63 in the best performing analysis (3). As a result, 3D-2 cm segmentations were selected for the following analyses. An overview of all feature-segmentation correlations for each segmentation approach is shown in Supplemental Table S1.

### Correlation analysis

**Figure 4:**
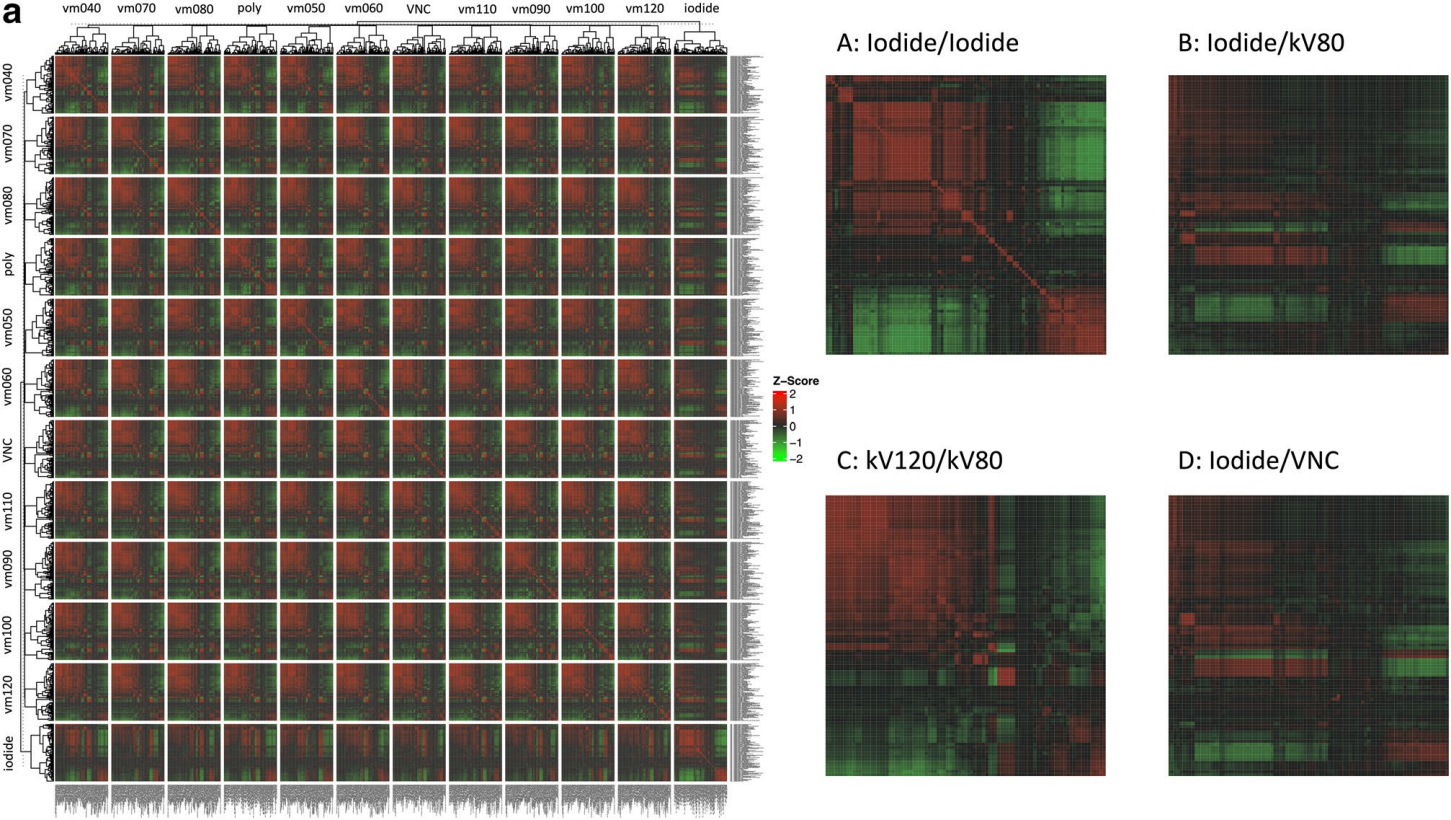

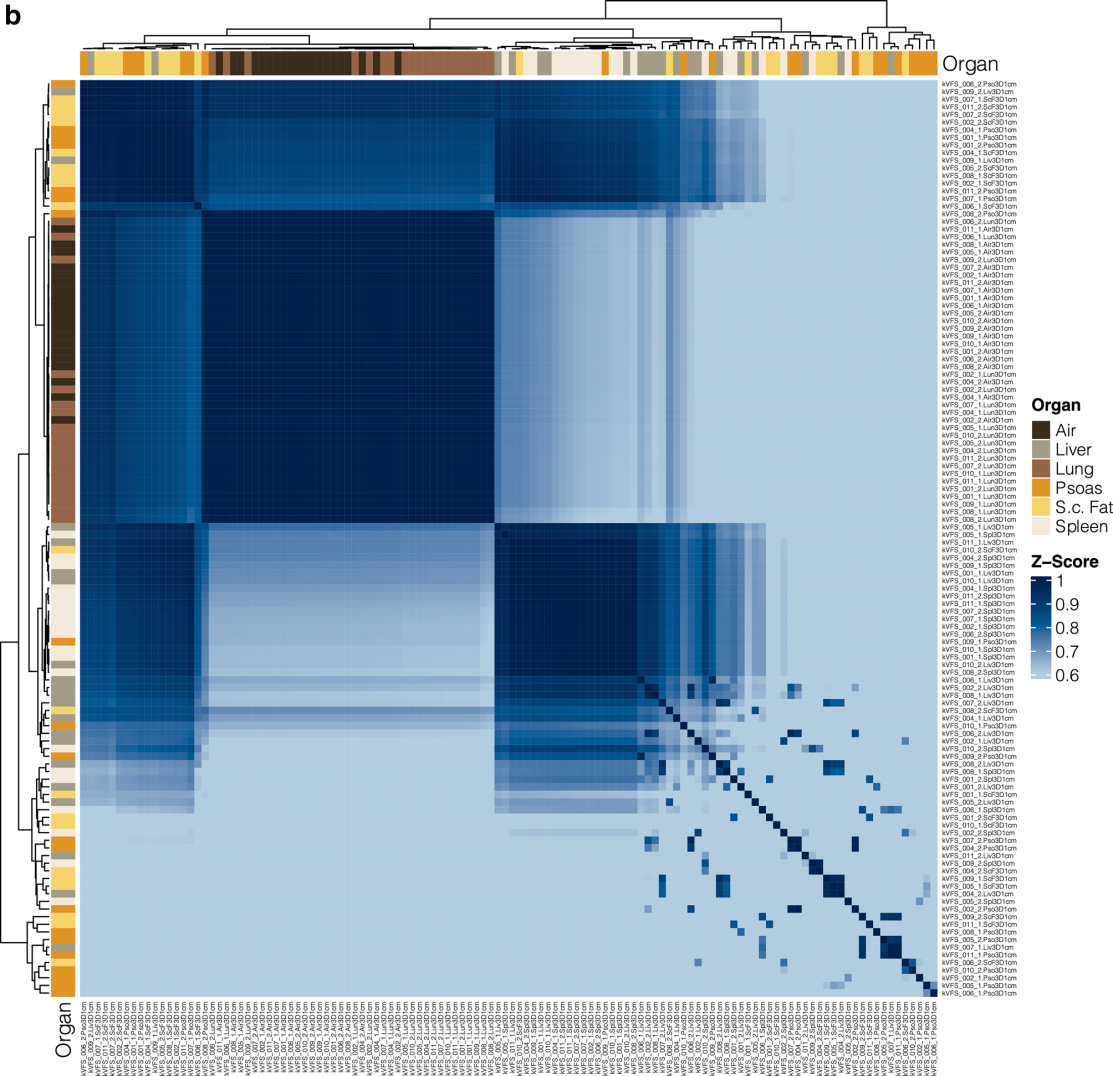
(**a**) Feature-wise correlation of reconstruction to reconstruction in 3D-2 cm split by reconstruction. (**b**) Segmentation to segmentation correlation based on 3D-2 cm.

A calculation of feature-wise correlation between reconstructions was performed and visualized in a correlation plot. This revealed a high degree of feature correlation within each reconstruction. Furthermore, hierarchical clustering resulted in a clustering of VM reconstructions together and an association of VNC with high energy VM-reconstructions. However, iodine map extracted features were clustered separately and had a distinctly lower degree of feature correlation with the other reconstructions (Fig. 4a).

In addition, an unsupervised organ-wise hierarchical clustering approach was performed to evaluate the presence of organ-specific feature patterns and their corresponding similarity. This revealed a high similarity of lung and air segmentations, and—to a slightly lower degree—spleen and liver segmentations (Fig. 4b).

### Identification of organ-specific stable features between monoenergetic reconstructions

The following analysis was performed on the best performing subgroup 3D-2 cm segmentation on only VM scans (3). Twelve features were identified as stable with an excellent ICC (> 0.75) across more than 3 organs in 3D-2 cm segmentations (Table 3). Mostly second-order texture features (glcm, gldm, glrlm, and ngdtm) were selected, but first-order feature entropy was also stable across different VM reconstructions. The relatively low degree of feature stability of first-order features, except first-order feature entropy, can be explained by the degree of variation of iodine contrast based on the monoenergetic level applied. A comparison of features with an excellent or good ICC level between different monoenergetic reconstructions showed a high level of feature stability in the lungs (93 stable features) and liver (69 stable features), while air (25 stable features) showed the lowest level of stable features.

**Table 3:** Stable features with ICC > 0.75 in > 3 organs between monoenergetic reconstructions for 3D-2 cm analyses.

	Air	Liver	Psoas	S.c. fat	Spleen
firstorder_entropy	0.43	0.76	0.90	0.76	0.82
glcm_Differenceentropy	0.40	0.83	0.76	0.75	0.89
glcm_Jointentropy	0.42	0.78	0.88	0.76	0.86
gldm_Dependenceentropy	0.45	0.89	0.92	0.78	0.84
gldm_DependenceNonUniformity	0.98	0.94	0.93	0.88	0.93
gldm_DependenceNonUniformityNormalized	0.44	0.95	0.93	0.88	0.92
gldm_GrayLevelNonUniformity	0.99	0.91	0.94	0.90	0.86
glrlm_GrayLevelNonUniformity	0.99	0.94	0.95	0.91	0.90
glrlm_RunLengthNonUniformity	0.93	0.89	0.86	0.84	0.90
glrlm_RunLengthNonUniformityNormalized	0.41	0.80	0.87	0.77	0.90
glrlm_ShortRunemphasis	0.51	0.83	0.92	0.79	0.90
ngtdm_Coarseness	0.95	0.99	0.96	0.98	0.98

### Analysis of segmentation-resegmentation stability by CCC

**Figure 5:**
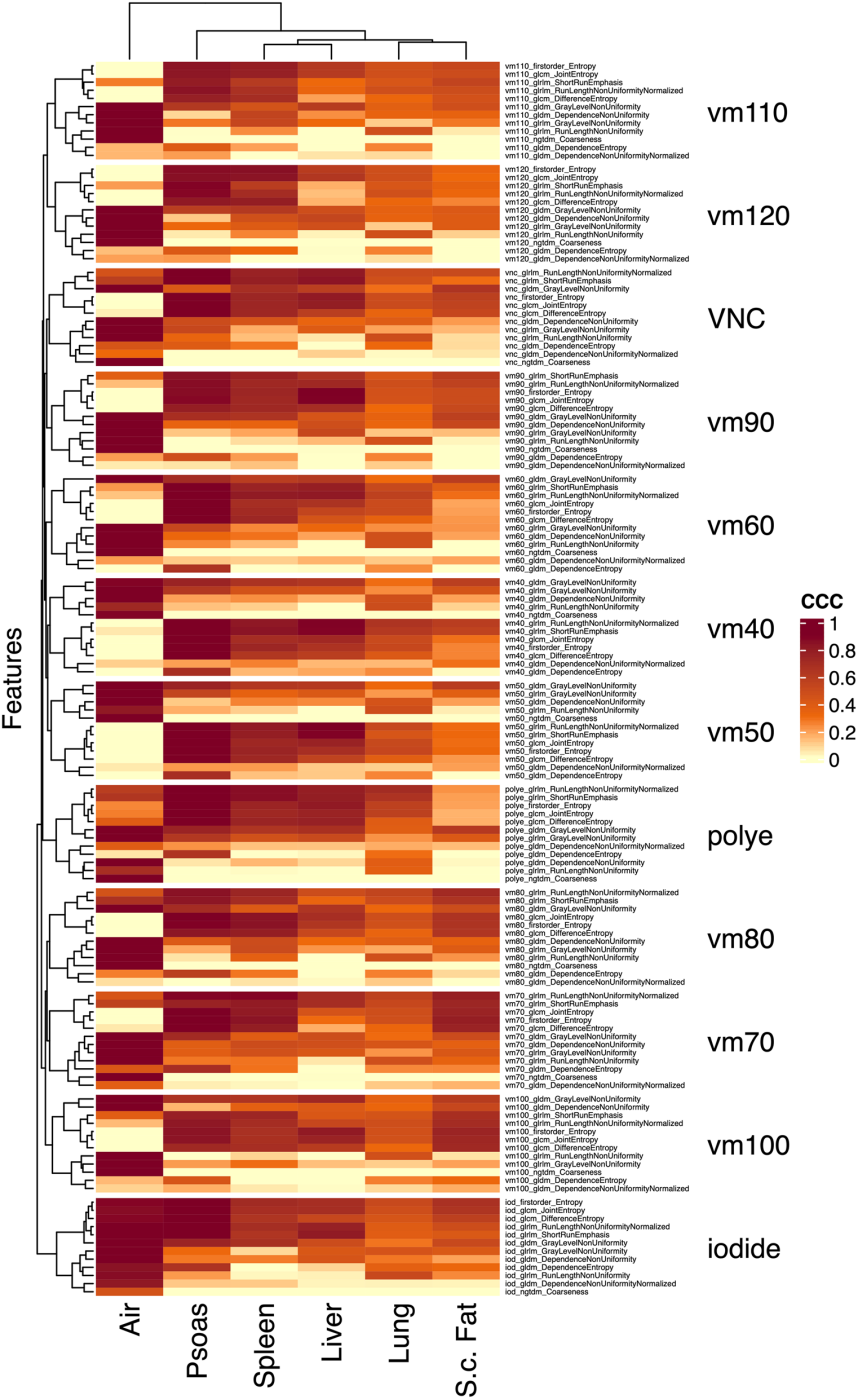
Concordance Correlation Coefficient for segmentation–resegmentation stability in 3D-2 cm segmentations, split by organ.

To validate the inter-segmentation stability of the identified features, Lin’s CCC for 3D-2 cm segmentations was calculated and plotted as a heatmap (Fig. 5). Overall, the CCC values of air differed significantly from all other regions. This may be due to the fact that air segmentations do not include material with relevant X-ray absorption. Specifically, segmentation-resegmentation stability was defined as a CCC above 0.6 in ≥ 3 organs in ≥ 6 VM reconstructions. This resulted in the further reduction of features and identification of the features glcm_JointEntropy, gldm_GrayLevelNonUniformity, and firstorder_Entropy. In summary, these features may be regarded as energy-independent and segmentation-resegmentation stable in this PCCT collective. The calculated CCC values can be found in Supplemental Material S3.

## Discussion

The goal of this study was the evaluation of radiomics feature properties across different virtual reconstructions in PCCT based on abdominal CT patient scans in the portal-venous contrast phase. PCCT is an emerging technology for clinical practice and has yet to be analyzed, particularly with regard to its relevance for radiomics feature values. This study did investigate feature stability patterns among organs and VM reconstructions and did also evaluate the segmentation-resegmentation stability of the features identified. In summary, 3D-2 cm segmentations showed the highest degree of stability. The observation is in line with other studies, which have for example shown in pulmonary nodules, that radiomics features of smaller nodules are more dependent on image acquisition parameters^17^. This could be interpreted as smaller regions having higher vulnerability to fluctuations and being more unstable. In addition to other factors, this could be due to random factors and image noise.

The most stable features were second-order texture features, whereas first-order features—except firstorder_Entropy—were relatively unstable among VM reconstructions—a finding which may be expected due to the varying iodine contrast. The observation that feature stability differs between the organs could also be related to the difference in contrast agent perfusion. Yet, air cannot be evaluated and interpreted similarly to other organs as it only represents image noise. This may be interpreted as a sign of homogeneous measurement throughout the field of view. Based on feature selection and segmentation-resegmentation evaluation with additional organ segmentation, the features glcm_JointEntropy, gldm_GrayLevelNonUniformity and firstorder_Entropy were considered stable in multiple VM reconstructions. Therefore, these features may be promising targets for clinical radiomics analyses that aim to rely on reproducible and stable features. The generation of VM reconstructions in PCCT may also be an opportunity to ensure comparability and transferability between study sites. For example, it could be possible to define a keV level for certain diagnostic tools, which would enable particularly good differentiation and comparability.

The results presented are in line with previous studies. For example, it was shown by Jensen et al. that radiomics features are dependent on the ROI size and volume in both MRI and CT when compared in a phantom^34^. In their comparison by assessment of the overall concordance correlation coefficient, CT features were less stable than MRI features.

The organ dependency of radiomics features has been studied by Lee et al.^35^, who investigated whether features are universally applicable. For this purpose, they used a radiomics score model which was based on Cox-LASSO regression coefficients. The comparison of lung, kidney, and brain showed differences in the radiomics signature. Test–retest setting analysis in lung and rectal cancers by van Timmeren et al.^36^ showed different levels of feature stability for both diseases with lung cancer showing more reproducible features (446/542 vs 9/542 in rectal cancer). Due to the different types of test–retest intervals used in their study, the results must be interpreted cautiously but nevertheless provide some indications for organ specificity of feature stability.

A recent review of the literature on this topic by Zhao et al.^37^ shows a trend toward better reporting, with newer publications increasingly including image acquisition parameter reports and standardization strategies. In their study that assessed radiomics feature stability by simulating CT acquisitions with different images^38^, Flouris et al. were able to reproduce feature instability. Analysis of simulated CT acquisitions could further elucidate the understanding of radiomics feature stability in future studies.

In theory, radiomics analyses could be implemented in the clinical workflow and used for diagnostic purposes or to evaluate the therapy response. As radiomics analysis needs additional prior segmentation and an automated segmentation is not available for every organ and disease of interest, the manual process could be time-consuming. Even though radiomics analyses would, more importantly, strengthen the clinical importance of the radiologist, the current time consumption and lack of ready-made solutions is a hurdle for clinical implementation^39^.

The conducted study has some limitations. First, our study is of a small sample size. Therefore, the results of this study must be considered preliminary. Nevertheless, the inclusion of > 500,000 radiomics feature values resulting from multiple segmentations per patient partly compensates for this limitation. Furthermore, the inclusion of iodine maps in the analysis must also be considered as a potential limitation, as this reconstruction only includes contrast-medium perfused areas. As the gray values weren't standardized the comparison of first-order features between iodine maps and other reconstructions is restricted. This limitation was addressed by excluding the iodine maps and other non-VM reconstructions in the final identification of stable features. However, the results presented within this paper are in line with expectations based on the physical properties of the reconstructions applied: while iodine maps did show a low degree of correlation with the other reconstruction types, features did show a better agreement between VM reconstructions. Concerning the selection of organ segmentations in this study, the inclusion of air into the analysis may be discussed, as it measures the background noise rather than any texture information. This theoretical reasoning is reflected in the results by a high degree of segmentation–resegmentation stability of the air. Concerning other organ segmentation, this study was able to show a high degree of agreement between segmentations of the same organ. Furthermore, organs with a similar visual texture, like liver and spleen, were clustered together when compared to other organs.

To overcome the challenges in reproducibility and stability of radiomics features, potential first solutions have been identified using PCCT. Furthermore, standardization initiatives such as the image biomarker standardization initiative^40^ can facilitate increasing the comparability of quantitative imaging data as it was shown that extraction algorithms can have an influence on radiomics features. Deep learning-based reconstruction models can be used^41^ to standardize images and increase feature stability. Now, the implementation of PCCT with its intrinsic capability of spectral reconstructions may be regarded as an additional tool to address the issue of feature stability, which remains a relevant impediment to the clinical application of radiomics, by utilization of energy-specific reconstruction techniques.

## Supplementary Information

Supplementary Information.

## Data Availability

The datasets generated during and/or analysed during the current study are available from the corresponding author on reasonable request.

## Abbreviations

CCC: Concordance correlation coefficient
EICT: Energy integrating computed tomography
GLCM: Gray level co-occurence matrix
GLDM: Gray level dependence matrix
GLRLM: Gray level run length matrix
GLSZM: Gray level size zone matrix
ICC: Intraclass correlation coefficient
NGTDM: Neighbouring gray tone difference matrix
PCCT: Photon-counting computed tomography
ROI: Region of interest
VM: Virtual monoenergetic
VNC: Virtual non-contrast
