# Supplementary material for: Evaluation of radiomics feature stability in abdominal monoenergetic photon counting CT reconstructions
Source: Sci Rep. 2022 Nov 15;12:19594. doi: 10.1038/s41598-022-22877-8 (PMC9665022; doi:10.1038/s41598-022-22877-8)
Supplement: Supplementary file 1 — Supplementary Information. [file 41598_2022_22877_MOESM1_ESM.docx]

# **Supplementary Material**

**Supplementary Table S1:** Overview of all feature-segmentation correlations of each segmentation approach


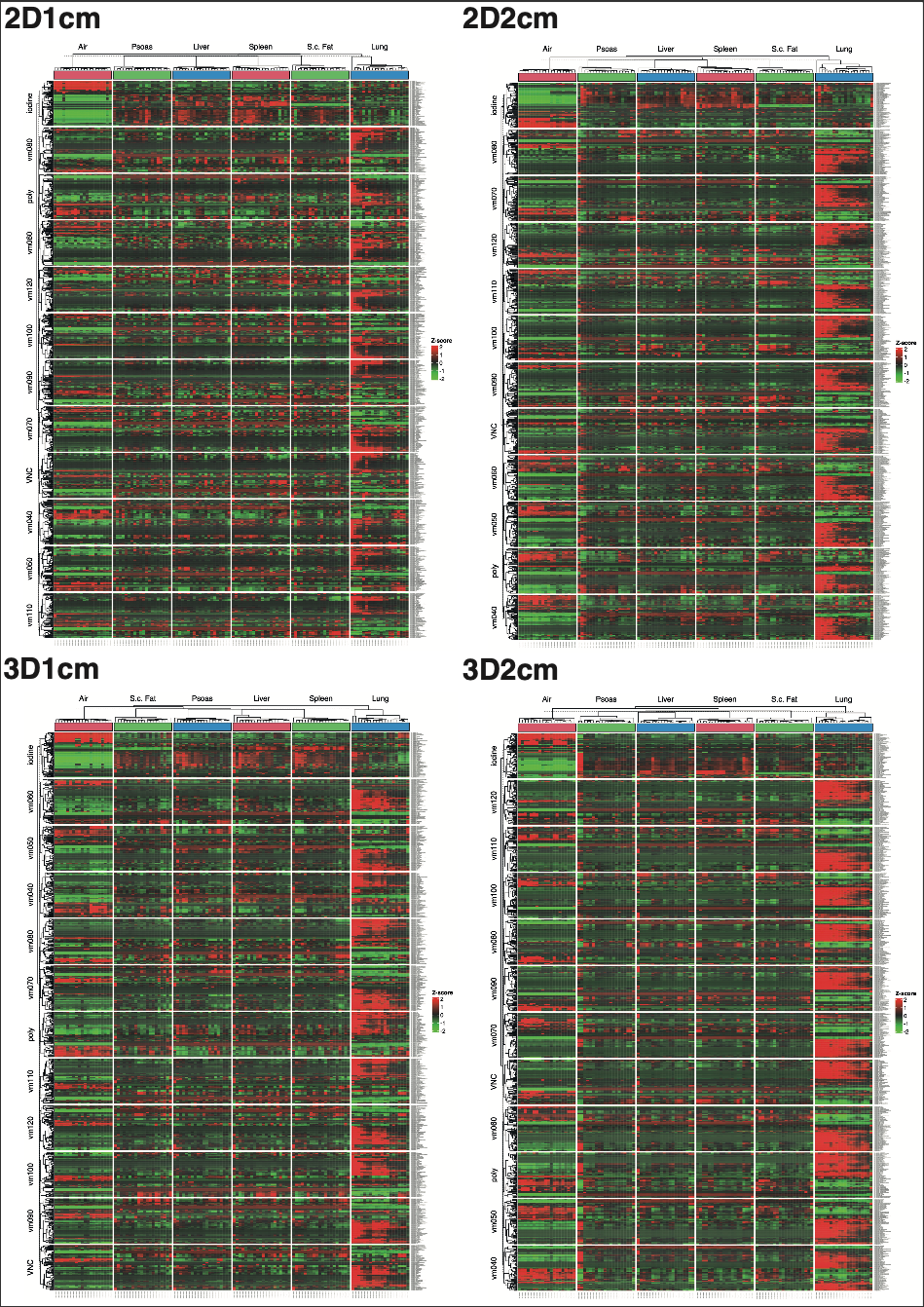


**Supplementary Table S2:** Radiomics extraction settings

Extraction parameters:

{'minimumROIDimensions': 2, 'minimumROISize': None, 'normalize': False, 'normalizeScale': 1, 'removeOutliers': None, 'resampledPixelSpacing': None, 'interpolator': 'sitkBSpline', 'preCrop': False, 'padDistance': 5, 'distances': [1], 'force2D': False, 'force2Ddimension': 0, 'resegmentRange': None, 'label': 1, 'additionalInfo': True}

Enabled filters:

{'Original': {}}

Enabled features:

{'firstorder': [], 'glcm': [], 'gldm': [], 'glrlm': [], 'glszm': [], 'ngtdm': [], 'shape': []}

**Supplementary Table S3:** CCC for all organs in 3D2cm

| Features | Air | Liver | Psoas | S.c. Fat | Spleen | Lung |
| --- | --- | --- | --- | --- | --- | --- |
| iod_firstorder_Entropy | 0.8828442 | 0.6917411 | 0.9818203 | 0.6689496 | 0.7093761 | 0.5072221 |
| iod_glcm_DifferenceEntropy | 0.8759445 | 0.5202622 | 0.9708433 | 0.5693721 | 0.6440026 | 0.4319422 |
| iod_glcm_JointEntropy | 0.8609336 | 0.6891788 | 0.9842456 | 0.643617 | 0.6854306 | 0.4746326 |
| iod_gldm_DependenceEntropy | 0.8448013 | 0.08639902 | 0.6544912 | 0.3157605 | -0.03612799 | 0.3511912 |
| iod_gldm_DependenceNonUniformity | 0.9273609 | 0.4330454 | 0.2709554 | 0.187346 | 0.2627377 | 0.2729845 |
| iod_gldm_DependenceNonUniformityNormalized | 0.8130902 | 0.0213837 | 0.1110427 | 0.02264515 | 0.10765 | 0.02633728 |
| iod_gldm_GrayLevelNonUniformity | 0.994878 | 0.4876968 | 0.7512342 | 0.510181 | 0.6795601 | 0.2782726 |
| iod_glrlm_GrayLevelNonUniformity | 0.9919031 | 0.4174025 | 0.3057602 | 0.4096142 | 0.07734579 | 0.4397882 |
| iod_glrlm_RunLengthNonUniformity | 0.8620233 | 0.02901577 | 0.2053386 | 0.2370182 | 0.01636822 | 0.5102509 |
| iod_glrlm_RunLengthNonUniformityNormalized | 0.9165421 | 0.7684601 | 0.9770149 | 0.4839447 | 0.6925284 | 0.3838372 |
| iod_glrlm_ShortRunEmphasis | 0.9241094 | 0.8056212 | 0.9586497 | 0.4103103 | 0.6410169 | 0.3729502 |
| iod_ngtdm_Coarseness | 0.4444447 | -0.0091718 | -0.04667594 | -0.007663612 | -0.02464907 | -0.002964667 |
| polye_firstorder_Entropy | 0.2422203 | 0.8080562 | 0.9671552 | 0.1871748 | 0.7506612 | 0.5652571 |
| polye_glcm_DifferenceEntropy | 0.3793296 | 0.7688363 | 0.9839146 | 0.1589291 | 0.7534542 | 0.3819245 |
| polye_glcm_JointEntropy | 0.2588253 | 0.7563643 | 0.9727343 | 0.1538135 | 0.7257337 | 0.5672414 |
| polye_gldm_DependenceEntropy | 0.05343912 | 0.01587948 | 0.6425415 | -0.06421645 | 0.00177616 | 0.2914938 |
| polye_gldm_DependenceNonUniformity | 0.9870952 | 0.08312764 | 0.05157363 | 0.01357929 | 0.1436812 | 0.3751662 |
| polye_gldm_DependenceNonUniformityNormalized | 0.3781236 | 0.1218257 | 0.2211426 | 0.1350184 | 0.1244998 | 0.1659521 |
| polye_gldm_GrayLevelNonUniformity | 0.9488131 | 0.6738007 | 0.7568795 | 0.6111112 | 0.6452617 | 0.3700749 |
| polye_glrlm_GrayLevelNonUniformity | 0.9696965 | 0.5053845 | 0.6221067 | 0.403959 | 0.4583757 | 0.2194322 |
| polye_glrlm_RunLengthNonUniformity | 0.6833078 | -0.04829289 | -0.1428176 | -0.1113038 | 0.008725185 | 0.3594123 |
| polye_glrlm_RunLengthNonUniformityNormalized | 0.5847405 | 0.7847472 | 0.9482826 | 0.2211441 | 0.8599049 | 0.7032308 |
| polye_glrlm_ShortRunEmphasis | 0.6363768 | 0.7948108 | 0.9366119 | 0.1952462 | 0.8588321 | 0.6540286 |
| polye_ngtdm_Coarseness | 0.919313 | -0.01086484 | -0.01836021 | -0.01375002 | -0.01463017 | -0.0189753 |
| vm100_firstorder_Entropy | -0.06493179 | 0.8063949 | 0.8375339 | 0.7509531 | 0.6716875 | 0.46064 |
| vm100_glcm_DifferenceEntropy | -0.06332415 | 0.6014734 | 0.7215857 | 0.7238511 | 0.6851321 | 0.3089784 |
| vm100_glcm_JointEntropy | -0.09772509 | 0.755224 | 0.8323932 | 0.7337158 | 0.6752169 | 0.4592336 |
| vm100_gldm_DependenceEntropy | 0.1383358 | -0.01496858 | 0.4275875 | 0.3096545 | -0.03798929 | 0.2583547 |
| vm100_gldm_DependenceNonUniformity | 0.9778413 | 0.4001582 | 0.1481228 | 0.5159788 | 0.3515508 | 0.3209104 |
| vm100_gldm_DependenceNonUniformityNormalized | 0.08999414 | 0.005778312 | 0.1629399 | 0.167498 | 0.04480318 | 0.07756623 |
| vm100_gldm_GrayLevelNonUniformity | 0.9772027 | 0.7256159 | 0.6588115 | 0.5996163 | 0.6453817 | 0.3555898 |
| vm100_glrlm_GrayLevelNonUniformity | 0.9887938 | 0.1273109 | 0.2013411 | 0.1878007 | 0.2919462 | 0.1003991 |
| vm100_glrlm_RunLengthNonUniformity | 0.9615679 | -0.09099357 | -0.07411906 | 0.05697462 | 0.08757993 | 0.4599624 |
| vm100_glrlm_RunLengthNonUniformityNormalized | 0.1369397 | 0.5138287 | 0.8220793 | 0.7019411 | 0.6962896 | 0.4779626 |
| vm100_glrlm_ShortRunEmphasis | 0.3747598 | 0.4122545 | 0.7738008 | 0.6805675 | 0.7399902 | 0.4389804 |
| vm100_ngtdm_Coarseness | 0.9274737 | -0.0270611 | -0.03425177 | -0.01320539 | -0.0166581 | -0.02295789 |
| vm110_firstorder_Entropy | -0.04699944 | 0.6159968 | 0.8335045 | 0.5176739 | 0.7858122 | 0.4657492 |
| vm110_glcm_DifferenceEntropy | -0.1329323 | 0.1909622 | 0.7762079 | 0.4533518 | 0.6969113 | 0.3143047 |
| vm110_glcm_JointEntropy | -0.09012564 | 0.6070782 | 0.8268486 | 0.4866364 | 0.7779186 | 0.4673596 |
| vm110_gldm_DependenceEntropy | 0.1447957 | -0.05745013 | 0.3894499 | -0.002806134 | 0.1884803 | 0.2487783 |
| vm110_gldm_DependenceNonUniformity | 0.9763136 | 0.2259191 | 0.08221491 | 0.3377791 | 0.5420704 | 0.3339393 |
| vm110_gldm_DependenceNonUniformityNormalized | 0.1453469 | 0.05435282 | 0.1984734 | -0.004180468 | -0.02013633 | 0.07224755 |
| vm110_gldm_GrayLevelNonUniformity | 0.9746783 | 0.6000093 | 0.6208699 | 0.4732799 | 0.4454199 | 0.3558505 |
| vm110_glrlm_GrayLevelNonUniformity | 0.986898 | 0.300785 | 0.2725596 | 0.2723701 | 0.5261956 | 0.103691 |
| vm110_glrlm_RunLengthNonUniformity | 0.953286 | -0.1139499 | 0.001799997 | 0.04280323 | 0.2318288 | 0.4677685 |
| vm110_glrlm_RunLengthNonUniformityNormalized | -0.02582152 | 0.3276704 | 0.8425477 | 0.4864469 | 0.6684798 | 0.4694628 |
| vm110_glrlm_ShortRunEmphasis | 0.2637526 | 0.3031813 | 0.7972103 | 0.5420277 | 0.6075747 | 0.4243534 |
| vm110_ngtdm_Coarseness | 0.906128 | -0.01169286 | -0.0397139 | -0.02406692 | -0.0005094719 | -0.02398981 |
| vm120_firstorder_Entropy | -0.03084104 | 0.5977929 | 0.8591928 | 0.3237077 | 0.8553862 | 0.4700938 |
| vm120_glcm_DifferenceEntropy | -0.1229636 | 0.1527519 | 0.8148022 | 0.2476367 | 0.7884974 | 0.3149934 |
| vm120_glcm_JointEntropy | -0.06482579 | 0.629298 | 0.8545827 | 0.2984183 | 0.8408901 | 0.4735947 |
| vm120_gldm_DependenceEntropy | 0.1312024 | 0.003775821 | 0.3986113 | -0.1993296 | 0.3070119 | 0.2525479 |
| vm120_gldm_DependenceNonUniformity | 0.9750458 | 0.5304777 | 0.1125736 | 0.3879725 | 0.4626263 | 0.3392027 |
| vm120_gldm_DependenceNonUniformityNormalized | 0.1902799 | -0.01844457 | 0.2065289 | -0.04521379 | -0.02552446 | 0.06301906 |
| vm120_gldm_GrayLevelNonUniformity | 0.9722955 | 0.4734888 | 0.5852293 | 0.4033814 | 0.6192602 | 0.3501419 |
| vm120_glrlm_GrayLevelNonUniformity | 0.9853222 | 0.4677251 | 0.3104606 | 0.3893569 | 0.3884203 | 0.10329 |
| vm120_glrlm_RunLengthNonUniformity | 0.9477195 | 0.02406967 | 0.04191294 | 0.0958543 | 0.2474627 | 0.4627089 |
| vm120_glrlm_RunLengthNonUniformityNormalized | -0.05156183 | 0.1303687 | 0.8758797 | 0.3119242 | 0.7024889 | 0.4659285 |
| vm120_glrlm_ShortRunEmphasis | 0.1996577 | 0.1598544 | 0.8841045 | 0.3414714 | 0.5922391 | 0.422436 |
| vm120_ngtdm_Coarseness | 0.8912294 | -0.006417189 | -0.03809292 | -0.02150432 | -0.02346673 | -0.02446666 |
| vm40_firstorder_Entropy | -0.2831937 | 0.6551423 | 0.9768001 | 0.2478204 | 0.7468563 | 0.5148236 |
| vm40_glcm_DifferenceEntropy | -0.0223652 | 0.5869222 | 0.9893626 | 0.247732 | 0.6320361 | 0.4010553 |
| vm40_glcm_JointEntropy | -0.2486502 | 0.7248963 | 0.98627 | 0.2848655 | 0.6947854 | 0.5017019 |
| vm40_gldm_DependenceEntropy | -0.3535154 | 0.1783163 | 0.6814225 | 0.01106611 | 0.1434889 | 0.2347881 |
| vm40_gldm_DependenceNonUniformity | 0.9700281 | 0.1543504 | 0.1898599 | 0.1887359 | 0.2256078 | 0.4717662 |
| vm40_gldm_DependenceNonUniformityNormalized | 0.09702098 | 0.1412443 | 0.1945422 | 0.2848026 | 0.2531256 | 0.1399568 |
| vm40_gldm_GrayLevelNonUniformity | 0.884608 | 0.6325571 | 0.7561825 | 0.5784567 | 0.6589567 | 0.290937 |
| vm40_glrlm_GrayLevelNonUniformity | 0.9627996 | 0.440217 | 0.6216365 | 0.439082 | 0.4420898 | 0.2314845 |
| vm40_glrlm_RunLengthNonUniformity | 0.7298266 | -0.03937454 | 0.1251722 | 0.09202782 | 0.09859086 | 0.48644 |
| vm40_glrlm_RunLengthNonUniformityNormalized | -0.01459365 | 0.8958788 | 0.9734413 | 0.5925287 | 0.8189166 | 0.6498458 |
| vm40_glrlm_ShortRunEmphasis | 0.04622675 | 0.883834 | 0.96477 | 0.551948 | 0.8404411 | 0.6113349 |
| vm40_ngtdm_Coarseness | 0.8896136 | -0.01306244 | -0.02530556 | -0.02094472 | -0.02003394 | -0.02679782 |
| vm50_firstorder_Entropy | -0.231434 | 0.6861544 | 0.970684 | 0.3086662 | 0.7545032 | 0.5258681 |
| vm50_glcm_DifferenceEntropy | -0.02985851 | 0.5513573 | 0.9868056 | 0.2094622 | 0.7349691 | 0.3616106 |
| vm50_glcm_JointEntropy | -0.1956689 | 0.7631507 | 0.9803152 | 0.2929755 | 0.7325426 | 0.5125249 |
| vm50_gldm_DependenceEntropy | -0.3005494 | 0.1102805 | 0.685846 | -0.0343437 | 0.1251145 | 0.245568 |
| vm50_gldm_DependenceNonUniformity | 0.9596325 | 0.2361609 | 0.1084892 | 0.1925005 | 0.2507453 | 0.4403073 |
| vm50_gldm_DependenceNonUniformityNormalized | 0.04240307 | 0.1071244 | 0.207604 | 0.1936612 | 0.2047964 | 0.09509555 |
| vm50_gldm_GrayLevelNonUniformity | 0.9552391 | 0.6115906 | 0.7559021 | 0.5973223 | 0.6229909 | 0.3037554 |
| vm50_glrlm_GrayLevelNonUniformity | 0.9748268 | 0.4111388 | 0.5344159 | 0.3621194 | 0.3446547 | 0.2071195 |
| vm50_glrlm_RunLengthNonUniformity | 0.813311 | -0.04676499 | 0.1622491 | 0.03725883 | 0.099744 | 0.4754813 |
| vm50_glrlm_RunLengthNonUniformityNormalized | -0.09866442 | 0.9128333 | 0.9578066 | 0.2959604 | 0.8021637 | 0.5112151 |
| vm50_glrlm_ShortRunEmphasis | -0.03505884 | 0.8968275 | 0.9326811 | 0.3064345 | 0.7882321 | 0.4288642 |
| vm50_ngtdm_Coarseness | 0.9389145 | -0.009486058 | -0.02476731 | -0.01609282 | -0.02072287 | -0.02403376 |
| vm60_firstorder_Entropy | -0.2986127 | 0.5352508 | 0.9657517 | 0.1920382 | 0.7172712 | 0.4951317 |
| vm60_glcm_DifferenceEntropy | -0.1020469 | 0.4383306 | 0.9725392 | 0.2111903 | 0.7365174 | 0.3431685 |
| vm60_glcm_JointEntropy | -0.278007 | 0.6595898 | 0.9779351 | 0.1829462 | 0.6897981 | 0.4935935 |
| vm60_gldm_DependenceEntropy | -0.03185227 | 0.008097689 | 0.6619905 | -0.1314227 | 0.002174893 | 0.2328806 |
| vm60_gldm_DependenceNonUniformity | 0.9578384 | 0.185105 | 0.3151046 | 0.1874927 | 0.2835836 | 0.4630106 |
| vm60_gldm_DependenceNonUniformityNormalized | 0.2045874 | 0.1531162 | 0.111011 | 0.1837779 | 0.0987362 | 0.1106509 |
| vm60_gldm_GrayLevelNonUniformity | 0.9850698 | 0.6210478 | 0.7046202 | 0.5894493 | 0.5911727 | 0.2993172 |
| vm60_glrlm_GrayLevelNonUniformity | 0.9864292 | 0.3133822 | 0.5261441 | 0.2185005 | 0.2406592 | 0.2273019 |
| vm60_glrlm_RunLengthNonUniformity | 0.911559 | -0.01773649 | 0.2422371 | -0.02399426 | 0.1439579 | 0.4668946 |
| vm60_glrlm_RunLengthNonUniformityNormalized | 0.1206083 | 0.8132785 | 0.9787576 | 0.2888627 | 0.8059632 | 0.5612282 |
| vm60_glrlm_ShortRunEmphasis | 0.2095634 | 0.7840318 | 0.9699592 | 0.3672946 | 0.7570543 | 0.5128396 |
| vm60_ngtdm_Coarseness | 0.9783696 | -0.015895 | -0.02440786 | -0.0158409 | -0.0183659 | -0.02208679 |
| vm70_firstorder_Entropy | -0.2333838 | 0.2966042 | 0.9406301 | 0.7839957 | 0.8377816 | 0.4729012 |
| vm70_glcm_DifferenceEntropy | 0.0439433 | 0.1638247 | 0.9103964 | 0.7565645 | 0.7943105 | 0.3181039 |
| vm70_glcm_JointEntropy | -0.2680542 | 0.4410477 | 0.9549414 | 0.757066 | 0.8083375 | 0.4746973 |
| vm70_gldm_DependenceEntropy | 0.4131616 | -0.0228131 | 0.68439 | 0.2455499 | 0.2835312 | 0.2391108 |
| vm70_gldm_DependenceNonUniformity | 0.9771066 | 0.4674982 | 0.3709071 | 0.3547116 | 0.487642 | 0.429113 |
| vm70_gldm_DependenceNonUniformityNormalized | 0.3649157 | -0.04910654 | 0.03454342 | 0.1493959 | 0.01734035 | 0.108724 |
| vm70_gldm_GrayLevelNonUniformity | 0.9873174 | 0.4809251 | 0.7137362 | 0.4933974 | 0.3808029 | 0.2960686 |
| vm70_glrlm_GrayLevelNonUniformity | 0.9914831 | 0.4170894 | 0.3717902 | 0.4156413 | 0.460092 | 0.2155684 |
| vm70_glrlm_RunLengthNonUniformity | 0.9519422 | 0.04863092 | 0.2730915 | 0.3433013 | 0.2794734 | 0.4684003 |
| vm70_glrlm_RunLengthNonUniformityNormalized | 0.4287486 | 0.7018805 | 0.8884029 | 0.7789562 | 0.8857754 | 0.5613665 |
| vm70_glrlm_ShortRunEmphasis | 0.5583804 | 0.6961616 | 0.7638891 | 0.7456231 | 0.8022641 | 0.5192787 |
| vm70_ngtdm_Coarseness | 0.982644 | 0.003967134 | -0.02436919 | -0.0259717 | -0.01357435 | -0.02007424 |
| vm80_firstorder_Entropy | -0.1910901 | 0.6385416 | 0.8989365 | 0.6479357 | 0.8443803 | 0.4653462 |
| vm80_glcm_DifferenceEntropy | -0.02456983 | 0.5152721 | 0.8263644 | 0.6421255 | 0.7924441 | 0.3277785 |
| vm80_glcm_JointEntropy | -0.233235 | 0.6862589 | 0.9132001 | 0.6541735 | 0.8337016 | 0.473949 |
| vm80_gldm_DependenceEntropy | 0.2587448 | -0.008587194 | 0.5791624 | 0.07865473 | 0.2750132 | 0.2556653 |
| vm80_gldm_DependenceNonUniformity | 0.9752433 | 0.2986923 | 0.39577 | 0.3377339 | 0.4875432 | 0.3710702 |
| vm80_gldm_DependenceNonUniformityNormalized | 0.06447142 | 0.01233125 | -0.03311179 | -0.003739849 | 0.05446823 | 0.106581 |
| vm80_gldm_GrayLevelNonUniformity | 0.9829503 | 0.6464556 | 0.6991729 | 0.4843407 | 0.3945514 | 0.3111264 |
| vm80_glrlm_GrayLevelNonUniformity | 0.9922266 | 0.2143918 | 0.1736315 | 0.3909491 | 0.5000107 | 0.1716367 |
| vm80_glrlm_RunLengthNonUniformity | 0.9640523 | -0.07285206 | 0.05566989 | 0.2181138 | 0.3525591 | 0.4654886 |
| vm80_glrlm_RunLengthNonUniformityNormalized | 0.4454771 | 0.4847162 | 0.8479303 | 0.717698 | 0.7486903 | 0.5383067 |
| vm80_glrlm_ShortRunEmphasis | 0.6620196 | 0.326113 | 0.8304912 | 0.652982 | 0.6999897 | 0.4937006 |
| vm80_ngtdm_Coarseness | 0.9712867 | -0.01204691 | -0.01796861 | -0.02457347 | -0.02252245 | -0.02401447 |
| vm90_firstorder_Entropy | -0.09865596 | 0.9101176 | 0.8623108 | 0.4929155 | 0.7422626 | 0.4532566 |
| vm90_glcm_DifferenceEntropy | -0.04092101 | 0.6974719 | 0.7853466 | 0.5117892 | 0.7263049 | 0.300273 |
| vm90_glcm_JointEntropy | -0.135138 | 0.9035402 | 0.8684854 | 0.4904333 | 0.7277033 | 0.4500622 |
| vm90_gldm_DependenceEntropy | 0.198387 | 0.0007861085 | 0.4709581 | -0.03627884 | 0.2002578 | 0.2489805 |
| vm90_gldm_DependenceNonUniformity | 0.9768987 | 0.4734998 | 0.3326377 | 0.519572 | 0.3328673 | 0.3228451 |
| vm90_gldm_DependenceNonUniformityNormalized | 0.04096613 | -0.03515225 | 0.05570237 | -0.1147954 | 0.1212625 | 0.1019368 |
| vm90_gldm_GrayLevelNonUniformity | 0.9793023 | 0.4335436 | 0.6807975 | 0.5706712 | 0.6489502 | 0.343366 |
| vm90_glrlm_GrayLevelNonUniformity | 0.9890789 | 0.5155882 | 0.1165212 | 0.1321496 | 0.1816448 | 0.1155983 |
| vm90_glrlm_RunLengthNonUniformity | 0.953111 | 0.1376639 | -0.09128373 | 0.0197097 | 0.06971911 | 0.4541278 |
| vm90_glrlm_RunLengthNonUniformityNormalized | 0.1342984 | 0.7294025 | 0.8631634 | 0.5578699 | 0.726424 | 0.4770923 |
| vm90_glrlm_ShortRunEmphasis | 0.3641393 | 0.6598394 | 0.8496312 | 0.5959685 | 0.7633099 | 0.4298358 |
| vm90_ngtdm_Coarseness | 0.9397699 | 0.003752508 | -0.02328917 | -0.02827531 | -0.04089431 | -0.02426938 |
| vnc_firstorder_Entropy | -0.2378341 | 0.8064442 | 0.9434362 | 0.5741156 | 0.7426142 | 0.4882963 |
| vnc_glcm_DifferenceEntropy | 0.03195834 | 0.5859177 | 0.9501138 | 0.5209655 | 0.7328267 | 0.3294626 |
| vnc_glcm_JointEntropy | -0.2746466 | 0.7948001 | 0.9349163 | 0.5529451 | 0.7374509 | 0.4996174 |
| vnc_gldm_DependenceEntropy | 0.4311004 | -0.05976757 | 0.3908353 | 0.07264817 | 0.2800278 | 0.3059039 |
| vnc_gldm_DependenceNonUniformity | 0.9727059 | 0.3279748 | 0.4876626 | 0.2046972 | 0.4354875 | 0.3844945 |
| vnc_gldm_DependenceNonUniformityNormalized | 0.3005783 | 0.09491588 | -0.03899427 | 0.04947467 | -0.04032097 | 0.009629153 |
| vnc_gldm_GrayLevelNonUniformity | 0.9874835 | 0.5787987 | 0.4110495 | 0.6525401 | 0.6892444 | 0.2867939 |
| vnc_glrlm_GrayLevelNonUniformity | 0.9901842 | 0.3880202 | 0.5198808 | 0.1414497 | 0.1658885 | 0.1841949 |
| vnc_glrlm_RunLengthNonUniformity | 0.9469933 | 0.05183711 | 0.3279468 | 0.06827308 | 0.1304946 | 0.4818947 |
| vnc_glrlm_RunLengthNonUniformityNormalized | 0.4514234 | 0.8125892 | 0.9789176 | 0.5064097 | 0.7610767 | 0.4941588 |
| vnc_glrlm_ShortRunEmphasis | 0.578425 | 0.8374406 | 0.9808501 | 0.2920095 | 0.7835406 | 0.4637408 |
| vnc_ngtdm_Coarseness | 0.9800419 | -0.0172268 | -0.01489356 | -0.02595447 | -0.02300325 | -0.02581566 |
